# Supplementary material for: Colistin Dosing Regimens against Pseudomonas aeruginosa in Critically Ill Patients: An Application of Monte Carlo Simulation
Source: Antibiotics (Basel). 2021 May 17;10(5):595. doi: 10.3390/antibiotics10050595 (PMC8157232; doi:10.3390/antibiotics10050595)
Supplement: Supplementary file 1 [file antibiotics-10-00595-s001.zip › antibiotics-1212075-supplementary.pdf]

Supplementary material

The Probability of target attainment (PTA) of different regimens at each minimum inhibitory concentration (MIC) level

Table S1. PTA of different regimens in CrCl range of 1-1 0 mL/min.

MIC (mg/L) Regimens	0.25	0.5	1	2	4	8	16	32	64
**LD 300 mg MD 60 mg q12h (120 mg) (EMA)**	100	99.96	99.49	95.24	75.36	38.35	9.88	0.77	0.04
**LD 300 mg MD 70 mg q12h (140 mg) (Nation)**	100	99.97	99.43	95	76	39.07	9.83	1.03	0.02
**LD 300 mg MD 80 mg q24h (80 mg) (our study)**	99.99	99.85	97.78	84.16	50.14	14.9	2.06	0.07	0
**LD 300 mg MD 33 mg q24h (33 mg) (our study)**	99.61	95.6	76.45	40.12	11.14	1.71	0.07	0	0
**LD 300 mg MD 40 mg q12h (80 mg) (our study)**	100	99.77	97.92	85.99	54.49	19.54	3.04	0.17	0
**LD 300 mg MD 66 mg q24h (66 mg) (our study)**	100	99.6	95.6	76.33	39.72	10.52	1.12	0.06	0

PTA: Probability of target attainment. CrCl: creatinine clearance. MIC: minimum inhibitory concentration. LD: loading dose (in mg of colistin base activity). MD: maintenance dose (in mg of colistin base activity). q12h: every 12 hours. EMA: European Medicines Agency. US-FDA: Food and Drug Administration of United State.

Table S2. PTA of different regimens in CrCl range of 11- 29 mL/min.

MIC (mg/L) Regimens	0.25	0.5	1	2	4	8	16	32	64
**LD 300 mg MD 80 mg q12h (160 mg) (Nation)**	99.95	99.71	98.45	90.59	65.99	29.9	5.83	0.4	0.01
**LD 300 mg MD 90 mg q12h (180 mg) (EMA)**	99.98	99.77	98.98	92.21	71.15	35.44	8.04	0.59	0.03
**LD 300 mg MD 90 mg q36h (60 mg) (US-FDA)**	99.67	97.01	78.55	39.17	9.83	0.8	0.02	0	0
**LD 300 mg MD 100 mg q24h (100 mg) (our study)**	99.86	98.98	93.85	74.37	37.3	8.72	0.7	0	0
**LD 300 mg MD 200 mg q36h (133 mg) (our study)**	99.95	99.78	97.84	83.31	44.13	12.2	1.18	0.04	0
**LD 300 mg MD 120 mg q24h (120 mg) (our study)**	99.94	99.39	96	81.28	46.78	13.97	1.63	0.07	0

**LD 300 mg MD 66 mg q24h (66 mg) (our study)**	99.62	97.33	84.64	52.72	17.86	2.55	0.16	0	0
**LD 300 mg MD 60 mg q12h (120 mg) (our study)**	99.93	99.54	96.44	82.8	51.08	17.86	2.55	0.1	0
**LD 300 mg MD 150 mg q24h (150 mg) (our study)**	99.96	99.76	97.9	88.35	59.5	22.19	3.6	0.11	0

PTA: Probability of target attainment. CrCl: creatinine clearance. MIC: minimum inhibitory concentration. LD: loading dose (in mg of colistin base activity). MD: maintenance dose (in mg of colistin base activity). q12h: every 12 hours. EMA: European Medicines Agency. US-FDA: Food and Drug Administration of United State.

Table S3. PTA of different regimens in CrCl range of 30- 50 mL/min.

MIC (mg/L) Regimens	0.25	0.5	1	2	4	8	16	32	64
**LD 300 mg MD 80 mg q8h (240 mg) (our study)**	99.74	98.95	96.16	86.15	62.54	29.01	6.85	0.61	0.01
**LD 300 mg MD 250 mg q24h (250 mg) (our study)**	99.76	99.18	96.61	85.6	58.65	24.96	4.6	0.29	0.02
**LD 300 ng MD 125 mg q12h (250 mg) (EMA)**	99.88	99.18	96.53	86.35	61.72	28.25	6.13	0.42	0.01
**LD 300 mg MD 110 mg q12h (220 mg) (Nation)**	99.67	98.71	94.9	83.11	55.45	22.83	4.21	0.3	0
**LD 300 mg MD 75 mg q12h (150 mg) (US-FDA)**	99.31	97.66	90.39	69.6	37.45	10.77	1.21	0.02	0
**LD 300 mg MD 120 mg q12h (240 mg) (our study)**	99.78	99.07	95.76	84.77	60.79	25.92	5.61	0.48	0.01
**LD 300 mg MD 100 mg q24h (100 mg) (our study)**	98.4	93.77	78.9	47.3	15.83	2.14	0.15	0	0
**LD 300 mg MD 100 mg q12h (200 mg) (our study)**	99.64	98.48	94.35	79.97	50.36	18.28	3.25	0.19	0
**LD 300 mg MD 200 mg q24h (200 mg) (our study)**	99.7	98.62	94.08	79.26	48.41	16.08	1.96	0.11	0

PTA: Probability of target attainment. CrCl: creatinine clearance. MIC: minimum inhibitory concentration. LD: loading dose (in mg of colistin base activity). MD: maintenance dose (in mg of colistin base activity). q12h: every 12 hours. EMA: European Medicines Agency. US-FDA: Food and Drug Administration of United State.

Table S4. PTA of different regimens in CrCl range of 51- 79 mL/min.

MIC (mg/L) Regimens	0.25	0.5	1	2	4	8	16	32	64
**LD 300 mg MD 150 mg q12h (300 mg) (EMA. Nation)**	98.93	96.52	89.99	75.02	47.33	18.87	3.46	0.28	0.01

**LD 300 mg MD 180 mg q12h (360 mg) (our study)**	99.22	97.21	92.44	79.6	55.2	24.87	5.96	0.58	0.01
**LD 300 mg MD 120 mg q8h (360 mg) (our study)**	99.29	97.3	92.32	80.15	56.64	27	7.07	0.83	0.04
**LD 300 mg MD 115 mg q12h (230 mg) (US-FDA)**	98.25	94.38	85.47	64.52	35.11	10.51	1.63	0.09	0
**LD 300 mg MD 100 mg q12h (200 mg) (our study)**	97.99	93.7	82.82	59.61	29.48	7.66	0.8	0.03	0
**LD 300 mg MD 100 mg q8h (300 mg) (our study)**	98.99	96.72	90.4	75.88	48.87	20.57	4.56	0.49	0.02

PTA: Probability of target attainment. CrCl: creatinine clearance. MIC: minimum inhibitory concentration. LD: loading dose (in mg of colistin base activity). MD: maintenance dose (in mg of colistin base activity). q12h: every 12 hours. EMA: European Medicines Agency. US-FDA: Food and Drug Administration of United State.

Table S5. PTA of different regimens in CrCl range of 80- 100 mL/min.

MIC (mg/L) / Regimens	0.25	0.5	1	2	4	8	16	32	64
**LD 300 mg MD 100 mg q6h (400 mg) (our study)**	98.26	95.12	88.16	73.69	47.16	15.12	1.17	0.01	0
**LD 300 mg MD 150 mg q8h (450 mg) (our study)**	98.24	94.96	87.38	72.07	47.77	21.82	5.41	0.52	0.06
**LD 300 mg MD 180 mg q12h (360mg) (Nation)**	97.49	93.37	83.54	64.36	37.17	13.54	2.48	0.18	0
**LD 300 mg MD 150 mg q12 h (300 mg) (US-FDA. EMA)**	96.88	91.32	79.36	57.85	30.55	9.38	1.37	0.06	0
**LD 300 mg MD 120 mg q6h (480 mg) (our study)**	98.57	96.42	90.96	79.23	55.82	23.18	2.95	0.1	0
**LD 300 mg MD 100 mg q8h (300 mg) (our study)**	96.91	91.79	80.71	60.15	32.21	10.53	1.67	0.14	0

PTA: Probability of target attainment. CrCl: creatinine clearance. MIC: minimum inhibitory concentration. LD: loading dose (in mg of colistin base activity). MD: maintenance dose (in mg of colistin base activity). q12h: every 12 hours. EMA: European Medicines Agency. US-FDA: Food and Drug Administration of United State.

Table S6. PTA of different regimens in CrCl range of 101- 130 mL/min.

MIC (mg/L) / Regimens	0.25	0.5	1	2	4	8	16	32	64
**LD 300 mg MD 150 mg q12h (300 mg) (EMA. US-FDA)**	93.08	83.65	66.7	43.75	19.86	5.06	0.59	0.04	0

**LD 300 mg MD 180 mg q12h (360 mg) (Nation)**	94.55	87.1	72.02	49.99	24.42	7.14	0.89	0.02	0
**LD 300 mg MD 100 mg q6h (400 mg) (our study)**	95.88	90.93	80.25	60.55	31.35	6.82	0.31	0.02	0
**LD 300 mg MD 100 mg q4h (600 mg) (our study)**	97.65	94.6	88.26	74.72	50.7	21.45	3.03	0.13	0
**LD 300 mg MD 150 mg q6h (600 mg) (our study)**	97.83	94.62	87.51	74.6	50.27	19.63	2.1	0.07	0
**LD 300 mg MD 120 mg q6h (480 mg) (our study)**	96.93	93.02	84.19	66.91	39.46	11.63	0.83	0.01	0
**LD 300 mg MD 200 mg q12h (400 mg) (our study)**	95.35	88.91	76.04	55.05	28.84	9.08	1.63	0.11	0

PTA: Probability of target attainment. CrCl: creatinine clearance. MIC: minimum inhibitory concentration. LD: loading dose (in mg of colistin base activity). MD: maintenance dose (in mg of colistin base activity). q12h: every 12 hours. EMA: European Medicines Agency. US-FDA: Food and Drug Administration of United State.
